# Supplementary material for: Are carbon emissions trading and green financial instruments synergistic? -Comprehensive quantitative research based on content analysis
Source: PLoS One. 2024 Mar 7;19(3):e0298601. doi: 10.1371/journal.pone.0298601 (PMC10919601; doi:10.1371/journal.pone.0298601)
Supplement: S1 Data — (ZIP) [file pone.0298601.s001.zip › Data/地级市Z_F工作报告.zip!/╔╜╬≈/│ñ╓╬╩╨/└┤╫╘╣1⁄2╓┌║┼í╛┬φ┐╦╩2╛▌═°í┐_2016.docx]

各位代表：

现在，我代表市人民政府向大会报告工作，请予审议，并请市政协委员和其他列席人员提出意见。

一、“十二五”时期国民经济和社会发展回顾

“十二五”时期是我市发展极不平凡的五年。面对严峻复杂的经济形势，我们在省委、省政府和市委的坚强领导下，按照中央“五位一体”总体布局、“四个全面”战略布局和省委“五句话”总要求，全面落实“六大发展”，务实推进“五五战略”，主动适应把握引领经济发展新常态，聚焦稳增长、促改革、调结构、惠民生、防风险，迎难而上、砥砺奋进，各项工作稳中有为、稳中有进，经济社会发展取得新成效。

五年来，我们多措并举稳定经济增长，综合实力有了新提升。坚持项目建设“六位一体”工作机制，实行重大项目联审联批，共实施重点项目2720个，全社会固定资产投资累计达到5328亿元，年均增长23.3%。引进外来投资项目1474个，实际到位资金2745亿元，是“十一五”时期的13.9倍。举办了首届长治制造展销推介周活动，鼓励本地企业在同质同价的条件下优先使用本地产品。认真落实企业减负政策，为企业减轻负担40多亿元。全市地区生产总值由2010年的920.2亿元增加到1195.1亿元，年均增长7.1%；一般公共预算收入由77.9亿元增加到96.44亿元，年均增长4.4%；社会消费品零售总额由272.6亿元增加到524.4亿元，年均增长13.2%。

五年来，我们坚定不移推进产业结构优化升级，转型发展迈出新步伐。大力发展现代农业，五年新发展设施蔬菜51.4万亩，总面积达到56.7万亩，是“十一五”末的10.7倍，发展速度连续五年全省第一，全市粮经作物种植比例由8:2优化为7:3；农产品加工龙头企业销售收入由70.8亿元增加到191.4亿元，年均增长22%。全力做好煤与非煤两篇文章，65座技改矿井已有30座竣工投产，煤炭就地转化率由30%提高到40%左右；围绕七大新兴产业，实施了高科LED、潞安太阳能等一大批新兴产业项目，新兴产业投资占工业总投资的比重由34%提高到57%；非煤产业增加值占工业增加值的比重由28.2%提高到33.1%。加快发展服务业，太行山农产品物流园、紫坊经贸物流园等4个较大规模的物流园建成运行；完成了太行山大峡谷资源整合，全市旅游总收入由88.8亿元增加到320.9亿元，年均增长29.3%，第三产业占地区生产总值的比重提高了12.9个百分点。全市三次产业结构由4.4:64.5:31.1调整为4.9:51.1:44。

五年来，我们狠抓城乡基础设施建设，城乡面貌发生新变化。市政基础设施建设累计完成投资210亿元，是“十一五”时期的2.2倍；城市道路总里程达到1004公里，比“十一五”末增长25%。特别是近三年来，在主城区开工建设各类城建重点工程438项，完成拆迁120多万平方米，是前十年总量的2倍，新敷设各类管道580多公里，新增道路面积150万平方米，初步形成了“三环八纵十二横”路网框架。体育中心、新闻中心建成使用。各县市区共实施城乡基础设施建设项目757项，完成投资208亿元，市区至6个卫星县城快速连接线基本贯通，53个重点镇和240个中心村建设步伐加快。全市常住人口城镇化率由41.84%提高到50.02%，年均提升1.64个百分点。全面完成城乡人居环境改善“四大工程”的各项任务。全市累计新增公路通车里程760公里，长治机场通航城市达到14个。

五年来，我们切实保障和改善民生，人民生活质量得到新提高。全市公共财政用于民生支出累计达到907亿元，年均增长14.8%。累计新增就业23.4万人，转移农村劳动力22.8万人。城镇居民人均可支配收入由15675元提高到26407元，年均增长11%；农村居民人均可支配收入由6032元提高到11095元，年均增长13%，城乡居民收入增速持续快于地区生产总值增速，农村居民收入增幅连续6年快于城镇居民收入增幅。探索建立了联盟校办学模式，实施了农村义务教育阶段寄宿制学生免费营养餐工程，主城区3所公办幼儿园全部建成。县级公立医院综合改革实现全覆盖，年人均基本公共卫生服务经费由25元提高到40元。开展出生缺陷干预救助“健苗工程”，为7.9万名新生儿进行了免费筛查。城乡居民养老、医疗保险实现全覆盖。累计开工建设各类保障性住房8.5万套，基本建成5.4万套。开工改造棚户区4.8万户，建成2.8万套，3.6万棚户区居民乔迁新居。实施易地扶贫搬迁5.28万人，共有19.34万贫困人口稳定脱贫。大力实施文化惠民工程，连续5年蝉联山西省公共文化服务体系绩效考核第一名。安全生产事故起数和死亡人数连续保持“双下降”态势。狠抓食品药品安全。开展社会治安“四个专项战役”，群众安全感和满意度名列全省前茅。

五年来，我们不遗余力改善生态环境，生态文明建设取得新成效。深入开展大气污染防治，2015年主要污染物PM10、PM2.5浓度分别比2013年实施新标准以来下降13.1%和19%，主城区二级以上天数增加60天，达到242天。开展焦化、电力、钢铁行业对标升级改造，累计淘汰落后产能1396万吨，万元地区生产总值综合能耗和“4+2”主要污染物减排任务全面完成。深入推进漳河、沁河流域生态环境综合治理，主城区应急备用水源主体工程基本完工，Ⅲ类以上水质断面达标率超额完成省定目标，辛安泉饮用水源达标率100%。累计完成造林210余万亩，森林覆盖率达到30.9%，比“十一五”末提高4.1个百分点。完成水土流失治理面积135万亩，治理度达到62%，名列全省第一。

五年来，我们持之以恒深化改革创新，经济发展增添新动力。在全省率先建成技术交易市场和技术交易信息服务平台，完成技术合同交易额16.2亿元，是“十一五”时期的2.2倍；专利申请量达到6738件，是“十一五”时期的2.9倍。建立政银企联席会议制度，设立了企业应急周转保障金，民间融资服务、股权托管、金融信息服务、应收账款交易等平台正式运行；成功通航在新三板挂牌；长治商业银行改制为长治银行，全市各类从事金融业务的机构发展到502家，2015年金融业实现增加值77.3亿元，是“十一五”末的2.6倍。实现“先照后证”，推行“三证合一”、“一照一码”，新发展私营企业1.7万户，年均增长23.1%，各类市场主体达到13.9万户，比“十一五”末增长67.4%；民营经济增加值占地区生产总值比重由32.8%提高到47.8%。建成院士、博士工作站33个，长治高新区升级为国家级高新区，长治海关正式批准设立，与韩国光州市结为友好城市。深入实施转型综改三年实施方案和年度行动计划，行政审批、土地管理、国企改制、教育、卫生、文化等各项改革顺利推进。

五年来，我们扎实推进民主法治和政府自身建设，依法行政又有新进步。认真执行人大及其常委会的决议、决定，积极支持人民政协履行职能，人大代表建议、政协委员提案办复率100%。加强与各民主党派、工商联、无党派人士和人民团体的联系，广泛听取各方面的意见和建议。全面完成“六五”普法任务。深入推进“六权治本”，实行“两清单、两张图、两平台、两监督”权力运行机制，建成启用新的政务服务中心。扎实开展党的群众路线教育实践活动、“三严三实”专题教育和学习讨论落实活动。严格履行政府系统党风廉政建设主体责任，狠刹“四风”，严惩腐败。“三公”经费持续下降。农村集体“三资”管理专项清理整治取得积极成效。实行市长县长电台接听制度，解决了一大批涉及群众切身利益的问题。

五年来，我们相继荣获全国文明城市、国家森林城市、国家现代农业示范区、国家公共文化服务体系示范区、全国全民健身示范市、国家智慧城市试点市、中国曲艺名城等十多个国家级荣誉称号，长治的美誉度和知名度进一步提升。

2015年是过去五年中经济下行压力最大、改革发展稳定任务最重的一年。一年来，我们全面落实省委、省政府和市委的决策部署，突出重点促发展，多措并举稳增长，盯住难点抓改革，勇于创新增活力，关注热点保民生，兜住底线增福祉。经过全市上下共同努力，经济运行中积极因素不断积累，“稳”的基础不断巩固，“进”的态势逐步向好，经济社会发展在十分困难的情况下负重前行。全年固定资产投资增长15.7%，社会消费品零售总额增长6%，城镇居民人均可支配收入增长7.5%，农村居民人均可支配收入增长7.6%。地区生产总值增长-2.9%，一般公共预算收入增长-29.26%，总量仍保持全省前三。

各位代表，过去五年取得这样的成绩实属不易，这是市委统揽全局、科学决策的结果，是市人大、市政协大力支持、有效监督的结果，是全市广大干部群众同心同德、团结奋斗的结果。在此，我代表市人民政府，向辛勤奋战在各条战线上的广大干部群众，向各位人大代表、政协委员、离退休老同志，向各民主党派、工商联、无党派人士和各人民团体，向驻市部队、武警官兵、公安干警，向所有关心、支持、参与长治建设发展的社会各界朋友，表示崇高的敬意和衷心的感谢！

各位代表，“十二五”时期政府工作行之有效的思路和做法，我们要在今后的实践中继续坚持。但我们也清醒地看到，当前我市经济社会发展还存在不少困难、矛盾和问题。一是经济持续下行压力还没有得到根本改观，部分企业生产经营困难，企业盈利能力普遍减弱，财政收支矛盾凸显，居民持续增收难度加大；二是经济结构调整任务艰巨，传统产业改造升级不快，新兴产业支撑作用不强，现代服务业相对滞后，农业基础依然薄弱；三是新的经济增长动能尚未完全形成，科技创新能力不强，资本市场发育不充分，民营经济发展质量不高，对外开放水平较低；四是社会民生事业欠账较多，城乡发展不均衡，脱贫攻坚任务艰巨，安全生产基础还不很牢固，生态环境形势依然严峻，就业、教育、卫生等工作还需加强；五是政府建设和干部作风与人民群众期盼还有差距，思想解放还不够，法治观念、服务观念、市场观念还不强，不作为、不会为、乱作为等问题仍在一定程度存在。因此，我们必须进一步增强忧患意识、责任意识、担当意识，全力以赴把政府工作做的更好。

二、“十三五”时期经济社会发展的目标任务

根据市委十届八次全会通过的“十三五”规划《建议》，我们编制了“十三五”规划《纲要（草案）》，提交本次大会审查。《纲要（草案）》按照紧紧围绕“四个全面”,牢固树立“五大理念”,统筹推进“六大发展”，确保如期实现脱贫攻坚和全面建成小康社会目标的要求，结合长治实际，分析了我市“十三五”时期面临的形势，提出了今后五年发展的指导思想、主要目标和任务。

今后五年我市经济社会发展的主要目标是：经济保持健康发展。到2020年地区生产总值和城乡居民人均可支配收入比2010年翻一番。城乡居民收入增幅不低于地区生产总值增幅，农村居民收入增幅不低于城镇居民收入增幅。经济结构明显优化。传统产业竞争力显著增强，新兴产业形成规模，服务业比重年均提高1个百分点以上，农业现代化水平持续提升。新型城镇化加速推进，常住人口城镇化率达到60%，户籍人口城镇化率达到43%以上。创业创新能力显著增强。创新要素配置更加高效，科技与经济深度融合，研究与试验发展经费投入强度达到2.5%，科技对经济增长贡献明显提高。大众创业、万众创新蓬勃发展，科教兴市和人才强市战略持续推进，发展新动能显著提升。人民生活水平和质量普遍提高。就业比较充分，基本公共服务均等化水平稳步提高。现行标准下农村贫困人口全部脱贫，贫困县全部摘帽。安全生产形势持续明显好转。全民素质和社会文明程度显著提高。社会主义核心价值观更加深入人心，诚信互助的社会风尚更加浓厚，公共文化服务体系基本建成，公民素质显著提高。生态环境质量明显改善。能源资源使用效率大幅提升，能源和水资源消耗、建设用地、碳排放总量得到有效控制，污染物减排完成国家、省下达任务。各方面制度建设更加完善。经济管理、行政管理和社会管理等重要领域和关键环节改革不断深化，体制机制更加完善。民主法治更加健全，法治政府基本建成。

各位代表，“十三五”规划《纲要（草案）》已经印发给大家，这里我重点对主要任务作一简要报告。

（一）全面落实创新发展，着力提高发展质量和效益。把创新作为引领发展的第一动力，抢抓供给侧结构性改革机遇，全力抓好“三去一降一补”各项工作。做好煤与非煤两篇文章，深入推进煤炭“六型”转变，加快冶金、电力、焦化等传统产业优化升级；积极发展现代煤化工、装备制造、新能源、新材料、中西制药、食品加工等新兴产业，培育一批新兴产业集群。大力发展现代农业，加快发展现代服务业，积极发展全域旅游，壮大文化旅游产业。实施“互联网+”行动，积极培育新兴业态，拓展发展新空间。加快转型综改试验区建设，全力推动科技创新、金融振兴、民营经济发展“三个突破”。

（二）全面落实协调发展，着力构建均衡发展格局。坚持发展经济和改善民生并重，促进经济社会协调发展。深入推进上党城镇群建设，大力实施城乡人居环境改善工程，加快城乡一体化发展步伐。全面完成棚户区、城中村改造任务。规划建设会展中心、文化艺术中心、科技馆、博物馆、图书馆、档案馆。壮大县域经济，促进区域协调发展。大力弘扬太行精神，繁荣文化事业，发展文化产业，推动物质文明和精神文明协调发展。支持国防和军队建设，促进军民融合发展。

（三）全面落实绿色发展，着力建设美丽长治。落实主体功能区定位，实行最严格的环境保护制度，深入推进大气、水、土壤污染防治行动。加强生态保护和修复，推进漳河、沁河流域生态环境综合治理。大力实施造林绿化，年均造林30万亩，力争森林覆盖率年均提高0.5个百分点。全面节约和高效循环利用资源，实行能源和水资源消耗、建设用地等总量和强度双控制，大力发展循环经济，打造低碳排放示范区。

（四）全面落实开放发展，着力打造内陆开放新高地。不断深化全方位开放合作，主动对接国家区域经济发展战略，积极参与“一带一路”建设。深化与东南沿海和中部地区的能源合作，加强与周边省、市的旅游开发合作。提高招商引资质量和水平，引进一批高新技术项目。加快对外贸易优化升级，完善外贸服务设施。创新发展“一区多园”模式，把高新区打造成对外开放的重要窗口和新的经济增长极。

（五）全面落实共享发展，着力保障和改善民生。坚持富民与强市相统一，扎实提高教育发展水平，积极扩大就业创业，多渠道增加城乡居民收入，健全完善社会保障制度。促进人口均衡发展，努力提高人民健康水平。坚决打赢脱贫攻坚战，确保到2020年现行标准下21.77万农村贫困人口全部脱贫，5个贫困县全部摘帽。

（六）全面落实廉洁和安全发展，着力创优发展环境。深入推进“六权治本”，严格落实政府系统党风廉政建设主体责任，完善“三重一大”决策机制，构建“亲”、“清”新型政商关系，营造廉洁发展环境。全面加强安全生产，夯实安全发展基础。健全公共安全保障体系，构建全民共建共享的社会治理格局，提升社会治理能力和水平。

各位代表，《纲要（草案）》经大会审议通过后，将成为我市“十三五”时期发展的宏伟蓝图。时代赋予我们责任，人民寄予我们厚望，只要我们咬定目标，同心同德，脚踏实地，真抓实干，就一定能够如期实现脱贫攻坚和全面建成小康社会目标！

2016年，我们要重点抓好以下几个方面工作：

（一）加强供给侧结构性改革，着力加快产业转型升级。

供给侧结构性改革是当前和今后一个时期经济工作的主线，是破解我市结构性困局，解决生产效率低下、产品档次不高、企业竞争力不强的难得机遇，我们必须抓住机遇、抓紧落实、抓出成效。

推进煤炭产业健康发展。认真落实全省煤炭供给侧结构性改革措施，生产矿井要严格按照276个工作日科学组织生产，基建矿井要加快建设，尽快形成优质产能，力争今年再有5座矿井投产达效，确保全市原煤产量稳定在1.1亿吨左右，其中地方煤矿5000万吨。加快煤炭综合信息平台和煤炭交易中心建设，积极拓展省外煤炭市场，鼓励市内企业就近使用本地煤炭，确保全市煤炭产销率达到90%以上。大力推动煤电、煤化、煤冶联营，构建煤-电-用产业链条，促进煤炭清洁高效利用。积极探索煤炭伴生资源开发利用新途径。

促进传统产业优化升级。积极帮助首钢长钢、兴宝钢铁等企业大力应用新技术、新工艺、新装备，降低生产成本，开发高附加值产品，提升产品竞争力。以潞安、潞宝等12户焦炭主体企业为基础，稳步推进现代化大机焦建设，全面提升焦炉煤气综合利用水平。推进现有电厂综合节能、超低排放标准改造，继续扩大大用户直供电规模，挖掘用电潜能，促进电力行业稳定增长。加快水泥、玻璃等行业的改造升级步伐。

大力发展新兴产业。以延伸现代煤化工产业链为重点，推进潞安180、瑞恒60万吨聚氯乙烯二期、潞宝己内酰胺等13个项目尽快投产达效，力争煤化工产业增加值增长5%。以煤机装备、汽车制造、数控装备、环保设备为重点，推进易通低温发电机组和脱硫脱硝设备、成功新能源汽车等12个项目尽快投产达效，力争装备制造业增加值增长5%。以新能源利用、新材料开发为重点，推进高科LED、平顺漳电光伏发电等22个项目尽快投产达效，力争新能源新材料产业增加值增长7%。以小杂粮、绿色蔬菜、核桃干果、农畜产品等特色优势产业为重点，培育壮大太行紫团、林盛果业、佰和园等龙头企业，力争农产品加工业增加值增长6%。以新型疫苗、生物制药、中药材为重点，推进康宝基因疫苗、振东50万亩连翘种植产业化等5个项目尽快投产达效，力争医药产业增加值增长8%。

大力发展文化旅游产业。推动全域旅游发展，完善旅游配套设施，抓好太行欢乐谷、平顺神龙湾、壶关桥上风情小镇等20个重点项目提档升级。建立全市旅游惠民“一卡通”，鼓励长治人游长治。积极推进壶关太行山大峡谷创建5A级旅游景区。加大旅游宣传推介力度。深入挖掘襄垣法显文化，依托“万里茶路”、“法显足迹”，开拓“一带一路”沿线旅游市场，积极拓展以韩国游客为主的海外市场，力争海外游客达到5万人次，旅游总收入增长15%以上。

大力发展现代服务业。统筹推进金融服务、信息服务、电子商务、检验检测等生产性服务业和家政服务、文化体育、健康养老等生活性服务业发展。规划建设城市商业综合体，提升现代服务业水平。进一步完善长治市物流产业发展规划，积极打造煤炭、农产品、中药材物流基地，促进中南铁路长子南集运站、黎城华驰物流等园区提升效益。加快发展第三方物流和多方物流，建立物流公用信息平台，打造物流产业链和产业集群。

积极发展新兴业态。实施“互联网+”行动，加强与移动、联通、电信、腾讯等互联网企业的合作，积极申报“宽带中国”示范城市，扎实推进“宽带长治”和“三网”融合建设，充分发挥信息化在经济社会发展中的“倍增器”作用，促进云计算、大数据、物联网等与传统产业、现代制造业和现代农业深度融合，不断催生新兴业态，发展分享经济。推进北斗数据中心、浪潮集团云计算等项目尽快建成运行。不断提升易淘、钜星、环渤海电子商务等孵化基地建设水平，努力形成电商企业集群发展态势。利用阿里巴巴、京东、乐村淘等知名电商平台发展农村电子商务。

（二）加快重点项目建设，保持经济平稳健康发展。

项目建设不仅能拉动当前的经济增长，又能夯实长远持续发展的基础。我们要加快发展，就必须有一批重大项目做载体、做支柱、做引擎。

加快推进重点项目建设。扎实开展“项目创新年”活动，进一步提高投资的有效性和精准性。今年初步安排重点工程项目942项，年计划完成投资700亿元。继续实行重点项目领导包联、联审联批制度，主动与省直部门对接，加快项目土地、规划、环评等手续办理。大力推广PPP模式，鼓励社会资本参与基础设施、公共服务设施等领域建设。大家期盼已久的太焦客专将于6月16日开工，今年还要重点推进4个低热值煤电厂、漳泽电力2×100万千瓦、长临高速、长治机场改扩建、207和208国道市区过境段改线等项目建设。

加大招商引资力度。充分发挥企业的招商引资主体作用，加大以企招商、以商招商工作力度。紧抓全省“民企入晋”重大招商引资活动的机遇，主动对接全国工商联110家常委企业、全国民营企业500强，每个县签约2-3个项目，力争全市实现签约1200亿元。抓住承接加工贸易产业转移的机遇，争取更多加工贸易企业落户我市。

提升对外开放水平。抢抓“一带一路”战略机遇，积极参加山西品牌“丝路行”、“中华行”等各项活动，帮助企业开拓中亚、欧洲等国际市场，完成外贸出口总额3249万美元。进一步密切与京津冀、环渤海经济圈和中原经济区的交流合作。开工建设长治海关及出入境检验检疫业务用房，积极争取将长治机场航空口岸建设列入国家口岸规划，力争实现临时开放。巩固发展与德国北威州玛尔市、韩国光州市等国际友好城市的经贸技术合作。

加大对实体经济帮扶力度。认真落实国家、省里的一系列扶持政策措施，积极争取上级支持，对困难企业逐一分析、一企一策，妥善解决职工就业、工资、社保等问题，努力降低企业综合成本，减轻企业负担。切实帮助民营企业解决资金、办理手续等方面的困难。稳妥处置“僵尸企业”。继续举办长治制造展销活动，支持企业走出去开拓市场、推广品牌、扩大销售。

（三）加快推进新型城镇化，促进城乡区域协调发展。

新型城镇化事关现代化建设全局，是一个既惠长远、也利当前的战略支点。我市正处在城镇化的快速发展阶段，必须按照中央城市工作会议“五个统筹”的要求，以城中村、棚户区改造为牵引，加快推进以人为核心的新型城镇化，让人民群众生活得更方便、更舒心、更美好。

继续推进主城区扩容提质。重点推进史家庄、马坊头、米家庄等21个城中村改造，开工改造城市棚户区1.2万户，基本建成各类保障性住房9000套。抓好“三河一渠”综合治理、火车站改造、湿地保护性开发等12项续建工程。开工建设改造府后西街、延安北路等14条市政道路、26条背街小巷、4座市政桥梁、4座人行过街天桥、2座铁路立交桥、9处停车场，同步推进道路配套管网和绿化工程。完成太行公园改造、城市公共自行车等项目。运用PPP模式，全力推进博物馆、图书馆、档案馆等项目建设。大力发展节能建筑，完成10万平方米既有建筑节能改造。加快供水、供热等城市基础设施建设，推进漳山电厂热电联供改造，完成辛安泉引水新建输水管道工程，启动辛安泉饮水水源升级改造、生活垃圾卫生填埋场、生活垃圾焚烧发电、污泥处置等项目。积极探索地下综合管廊和“海绵城市”建设。加快“智慧城市”建设步伐，完善城市综合管理系统，探索建立城市管理综合执法机制，全面提升城市精细化管理水平。

积极推进上党城镇群建设。以“核心产业支撑、快速交通连接、优美村镇点缀、都市农业衬托”为方向，积极推进“多规合一”，加快6个卫星县城与主城区的设施对接、产业对接、机制对接、服务对接，加强互联互通，实现资源共享。大力推广长子县城市建设经验，加快大县城建设，进一步完善各类基础设施，做大做强县域经济，提高县城对人口和产业的承载力。积极推进特色工业、生态旅游、物流交通、休闲商贸等各具特色的重点镇和中心村建设。

完善城镇化推进机制。深化户籍管理制度改革，有序推进农业转移人口市民化，确保户籍人口城镇化率再提高1个百分点以上。实施居住证制度，努力实现基本公共服务常住人口全覆盖。维护进城落户农民土地承包权、宅基地使用权、集体收益分配权，支持其依法有偿转让。加快形成以城带乡、城乡互惠的新型城乡关系。推进城乡基础设施一体化建设，实现水电路气等基础设施城乡互联、共建共享，构建城乡一体化发展格局。

积极化解房地产库存。加强房地产市场管控，落实好住房信贷、税收优惠政策，鼓励城镇居民改善住房条件，支持农村转移人口在城镇购房。打通商品房和公租房供需通道，将公租房保障范围由城镇户籍家庭扩大至城镇常住人口家庭。放宽住房公积金提取条件，提高住房公积金使用率。加大棚户区改造货币化安置力度，货币化安置比例达到50%以上。积极发展住房租赁市场。

（四）大力发展现代农业，扎实做好“三农”工作。

农业仍然是经济社会发展的短腿，农村仍然是实现全面小康的短板。我们必须坚持以发展特色现代农业为重点，以促进农民增收为核心，以深化农村改革为动力，全力实现农业强、农民富、农村美。

发展特色现代农业。大力推进国家现代农业示范区建设，努力创建全国优质玉米产业示范区和北方地区设施蔬菜样板区。重点抓好105个新建改扩建农业产业化项目，培育壮大30个农业产业化龙头企业，确保农业产业化龙头企业销售收入增长8%以上。推行农业标准化生产，稳步推进无公害、绿色、有机和地理标志农产品发展，新认证“三品一标”农产品50个。加强农产品质量安全监管。积极推广秸秆综合利用技术，加快推进主要农作物全程机械化。

千方百计促进农民增收。重点建设20个粮食千亩高产示范片，新发展设施蔬菜6万亩、干果经济林10万亩、小杂粮8万亩、中药材5万亩、油用牡丹5万亩，增加农民家庭经营性收入。培训新型职业农民8000名，促进农民就业创业，增加农民工资性收入。引导农户自愿以土地经营权等入股龙头企业和农民专业合作社，增加农民财产性收入。落实好各项强农惠农富农政策，确保各项补贴资金及时兑现到位，增加农民转移性收入。

深入推进农村人居环境改善。抓好165公里农村公路拓宽改造和318公里村通水泥（油）路完善提质工程，完成5346户采煤沉陷区治理搬迁、8380户农村危房改造、309户农村地质灾害治理搬迁。建设50个生态村庄，新建50个农村老年人日间照料中心。启动长子县农村垃圾治理示范县和平顺县农村生活污水治理试点县建设，完成5.77万户农村无害化卫生厕所改建任务。新启动12个省级、20个市级美丽宜居示范村创建工作。

深化农村综合改革。坚守耕地红线，全面完成基本农田划定并永久保护。稳定完善农村土地承包经营关系，基本完成农村土地承包经营权确权登记颁证工作，鼓励农民依法自愿有偿流转承包地，发展多种形式的适度规模经营，不断壮大新型农业经营主体。稳步推进国有林场改革，继续深化集体林权制度改革。积极推进襄垣、潞城、武乡、壶关4个省级试点县供销社改革。做好第三次全国农业普查工作。

（五）加快脱贫致富步伐，坚决打赢脱贫攻坚战。

农村贫困人口全部脱贫是习近平总书记代表全党向世界作出的庄严承诺。我们要以脱贫攻坚统揽经济社会发展全局，把脱贫攻坚作为“头等大事”和“第一民生工程”，下大力气啃下这块最难啃的硬骨头。

严格落实脱贫攻坚责任制。坚持“一把手”负总责，市里重点做好上下衔接、统筹协调、督促检查工作，县级承担主体责任，做好进度安排、项目落地、资金使用、人力调配、推进实施等工作。乡、村承担具体责任，做好贫困户精准识别、脱贫退出和资金、项目具体实施等各项扶贫工作，层层签订责任书，确保各项责任落实到位。

精准落实帮扶措施。坚持精准扶贫、精准脱贫，做到扶贫对象、项目安排、资金使用、措施到户、因村派人、脱贫成效“六个精准”。开展领导干部带头帮扶、机关单位包村帮扶、党员干部到户帮扶、工作队员驻村帮扶、第一书记任职帮扶“五帮联动”，因地制宜实施发展生产、易地搬迁、生态补偿、发展教育和社会保障“五个一批”工程，加快脱贫致富步伐。

全面落实重点任务。加快推进平顺、壶关、武乡3个县、4650户的光伏扶贫项目。完成9781人的易地扶贫搬迁任务。对1850名贫困人口进行就业培训，切实提高贫困劳动力的就业创业能力。建立健全脱贫攻坚考核机制、贫困县退出机制和第三方评估机制，确保年内5万贫困人口稳定脱贫、192个贫困村退出。

（六）持续推进民生改善，进一步提高人民生活水平。

民之所望，施政所向。任何时候任何情况下，我们都要始终坚持把广大人民的利益放在首位，千方百计把为民造福的事办实、办好。

着力稳定和扩大就业。全面落实创业就业扶持政策，不断深化创业型城市建设，继续对高校毕业生创业实行“七补一贷”，对小微企业实行“六补一缓”，推动大众创业、万众创新。提高公共就业服务水平，做好农村转移劳动力、城镇失业人员、退役军人等群体就业工作。强化技能培训，推行终身职业技能培训制度，实施职业培训全覆盖计划。认真落实降低社保费率等政策，加强失业预警监测工作，稳定就业岗位。健全农民工工资支付保障机制。

均衡发展教育事业。抓好主城区新建、改扩建8所义务教育学校工程，促进优质教育资源均衡布局，缓解义务教育入学紧张局面。巩固强化义务教育经费保障机制，实施好农村寄宿制学校免费营养餐工程。确保郊区、壶关通过义务教育基本均衡县验收。扩大学前教育资源，积极发展普惠性幼儿园，进一步规范幼儿园收费标准。加快普及高中阶段教育，主动适应高考改革。扎实推进职教园区、体校搬迁等项目建设，切实做好校企合作，努力提高职业教育发展水平。支持特殊教育健康发展。

提升医疗健康水平。继续深化医药卫生体制改革，稳妥推进城市公立医院改革，巩固县级公立医院改革成果，建立基层医疗卫生机构运行新机制。完善基本药物制度和公立医院药品采购机制。开工建设市医院、市二院二期工程，加快市妇幼保健院扩建步伐，推进儿童医院、精神病院建设，完成16个乡镇卫生院、876个村卫生所新建改造工程。大力推进优质医疗资源下沉，创新医疗联合体建设运营模式，全面实施分级诊疗。提升中医药服务能力。强化重大疾病防控。稳妥推进全面两孩政策实施，提高公共卫生计生服务质量。继续实施“健苗工程”，确保新生儿疾病筛查率达到95%以上。

完善社会保障体系。稳步推进机关事业单位、企业基本养老保险制度并轨运行，继续提高退休人员基本养老金待遇水平。建立统一的城乡居民基本医疗保险制度，年人均财政补助标准由380元提高到420元。完善重特大疾病医疗救助政策。落实经济困难的高龄与失能老年人补贴以及百岁老年人补贴新标准，城乡低保家庭中80到90岁的老年人每月补贴30元，60到90岁的失能老年人每月补贴60元，百岁以上老年人补贴每月不达300元的一律提高到300元。为困难残疾人每月发放50元生活补贴，为重度残疾人每月发放50元护理补贴。推进农村低保标准与国家扶贫标准相衔接。

大力发展文化事业。进一步巩固拓展国家公共文化服务体系示范区创建成果，实施基层公共文化服务提升工程，开展好周末大剧院等各类基层文化惠民活动。加快推进市梆子剧团、落子剧团、豫剧团、杂技团四个院团的排练场地建设，确保主体完工。推进乡村文化记忆工程，加大红色文化传承保护与开发力度。加强文物和非物质文化遗产保护。发展新闻出版、广播影视、文学艺术事业。倡导全民阅读。净化网络环境，加大“扫黄打非”力度。加强对外宣传和文化交流，推动传统媒体和新兴媒体融合发展。大力发展体育事业，做好全国体育产业联系点工作。

加强和创新社会治理。全面推进法治长治建设，启动实施“七五”普法，营造崇尚法治的良好社会氛围。加强公共法律服务体系建设，搞好法律援助。扎实做好信访工作，有效调处化解矛盾纠纷。深化“平安长治”建设。做好“五大领域”风险预警、预控工作，打好禁毒攻坚战。创新城乡基层群众自治和社区治理，加强对特殊人群的帮扶和管理，做好社区矫正工作。关爱农村留守儿童、妇女和老人，加强未成年人社会保护。健全完善信用体系，实现部门之间信用信息共享。强化市场监管，狠抓食品药品综合治理。

做好国防动员、国防教育、民兵预备役、双拥和人防工作，促进军民融合发展。进一步加强气象、地震、档案、外事侨务、民族宗教、对台、地方志等工作，关心支持妇女儿童、老龄、红十字会、慈善救助等事业。

（七）狠下功夫搞好节能减排，大力度推进生态文明建设。

绿水青山就是金山银山。我们必须全市齐动员、全民共行动，深入持久地推进生态文明建设，让上党大地天更蓝、水更清、山更绿。

突出大气污染防治。深入开展大气环境综合整治专项行动，全面完成主城区216台供热燃煤锅炉改造和54台经营性燃煤锅炉清洁能源替代，加快城中村土小燃煤锅炉淘汰和清洁能源替代步伐。完成26家焦化企业、5家钢铁企业提标改造。淘汰黄标车、老旧车1.1万辆。积极推广使用新能源汽车，新运营50辆新能源纯电动公交车。严格落实建筑工地、道路保洁、渣土运输和堆场作业扬尘控制措施，确保PM10、PM2.5浓度比上年分别下降4%和3%。

强化水、土壤污染防治。积极推进漳河、沁河流域水环境综合治理，加快建设郊区黄碾人工湿地，开工建设北寨人工湿地。加快石子河下游等3处黑臭水体治理。建立健全从水源到水龙头的全过程监管机制，确保饮用水安全。全面控制重点行业水污染物排放。加大土壤环境监测力度，加强农业面源污染防治，开展污染场地环境风险评估制度建设，明确保护、禁止、限制和控制的措施要求。

构筑生态安全屏障。全力实施林业“六大工程”，抓好太行、太岳“两山”和漳河、沁河“两河”流域生态脆弱区植被恢复，完成造林30万亩，完成水土流失治理24万亩。

创新生态环境保护机制。深入实施主要污染物排污权有偿使用和交易制度。大力推进环境污染第三方治理，按照政府引导、市场化运作、排污者付全费的原则，对污染物实施专业化治理。建立完善环境保护联合执法工作机制，严厉打击重点领域环境违法行为。加快建立完善生态环境监管、生态保护补偿、工作考核问责等制度体系。

（八）全面深化改革创新，激发全社会创造活力。

改革创新是发展的不竭动力和永续源泉。我们要始终把发展的基点放在改革创新上，深入实施创新驱动发展战略，全方位推进各个重点领域改革。

大力推进科技创新。把科技创新摆在更加重要位置，持续深化科技体制改革，着力破除制约科技创新的体制机制障碍。进一步完善长治技术交易市场服务体系，确保完成省下达的技术合同交易任务。强化企业创新主体地位和主导作用，支持创新型企业发展，新培育认定一批国家高新技术企业、省级民营科技企业和企业工程技术（研究）中心。继续强化政校企联合、产学研一体发展，引导社会资本参与科技创新项目。创新人才体制机制，激发本土人才创新创造活力，瞄准“高精尖缺”引进各类人才。加强科学普及教育，提高全民科技素质，让全市人民的创新智慧充分释放、创新力量充分涌流。

积极促进金融振兴。深化长治银行、农商银行、村镇银行等地方金融机构改革，加快黎城、沁县、沁源信用社改制步伐，加快推进金融商务区建设。不断完善金融服务体系，创新金融产品和融资模式，防范化解金融风险。充分发挥政府投资基金作用，吸引更多社会资本和金融资本，助推经济社会发展。实施企业上市培育工程，力争全年有1家企业在主板上市、10家企业在新三板挂牌。支持企业调整负债结构，尽快促成潞宝、襄矿分别发行10亿元企业债券。

加快发展民营经济。以建设全省民营经济试点市为契机，鼓励民营企业依法进入更多领域。抓好2个重点县、10个重点行业、50个重点企业试点，争创1个民营经济省级创业创新示范县。继续办好“长清班”，为民营企业发展培训更多管理人才。加快创业基地建设，新创办小微企业3000户以上，新培育“小升规”企业10户以上、“小巨人”企业6户以上，力争民营经济增加值占地区生产总值的比重提高2个百分点。

统筹推进各领域改革。围绕转型综改试验区建设，落实好国家、省部署的各项改革任务，制定实施转型综改“十三五”方案和2016年行动计划。深入推进国有企业和城镇集体企业改革，加快推进党政机关与所办企业脱钩改革。推进不动产登记工作。深化财税体制改革，加大预算统筹力度，扩大预算公开范围，清理、整合、规范专项转移支付，增加一般性转移支付规模和比例。依法实施税收征管，全力推进“营改增”、资源税改革。稳步推进工资收入分配制度改革、社会保障制度改革。

（九）毫不松懈抓好安全生产，着力夯实安全发展基础。

安全是一切工作的底线，是不可逾越的红线。我们要始终把人民生命财产安全放在首位，牢固树立安全发展理念，把安全生产责任落到实处。

落实安全生产责任。健全完善“党政同责、一岗双责、失职追责”和“管行业必须管安全，管业务必须管安全，管生产经营必须管安全”的安全生产责任体系，严格实施安全生产目标责任考核“一票否决”。切实履行安全生产监管责任，严格执行“四不两直”和“两议一报”制度，强化企业安全生产主体责任，加大安全生产约谈力度，以最严格的措施落实安全生产责任。

夯实安全生产基础。大力推进企业安全生产标准化建设，切实做到安全生产责任到位、投入到位、培训到位、基础管理到位和应急救援到位。建立完善风险管控和隐患排查治理双重预防机制。加强应急管理和防灾减灾能力建设，健全应急预案体系，强化应急救援培训，完善预警联动机制。

严格安全生产监管执法。持续开展全方位、立体式安全生产大检查，深入开展专家巡检会诊，全面加强各个行业各个领域的安全生产。按照“四不放过”原则，严肃查处事故，严格追究责任，坚决防控重特大安全生产事故，减少较大事故和一般事故，促进安全生产形势持续明显好转。

（十）切实加强政府自身建设，努力打造人民满意政府。

重任千钧惟担当。加强政府自身建设既是人民群众的殷切期盼，也是做好各项工作的坚实保障。我们必须牢固树立政治意识、大局意识、核心意识、看齐意识，努力建设人民满意的法治政府、创新政府、廉洁政府、服务型政府，推动政府治理体系和治理能力现代化。

全面推进依法行政。严守宪法和法律，自觉运用法治思维和法治方式推动工作，把政府的决策、执行以及日常管理全面纳入法治轨道。深入实施“六权治本”，切实规范行政权力运行。严格落实行政执法责任制，促进规范公正文明执法。加强政府法制和行政复议工作。自觉接受人大法律监督、政协民主监督、社会舆论监督，认真办理人大代表建议和政协委员提案，广泛听取各民主党派、工商联、无党派人士意见，积极支持工会、共青团、妇联等群众团体开展工作。

积极创新政府管理。坚决破除传统思维定势，进一步解放思想，努力在变中求新、新中求进、进中突破。建立健全激励机制和容错纠错机制，营造肯干事、能干事、干成事的浓厚氛围。坚决破除工作惯性，从墨守成规中走出来，从按部就班中走出来，不断创新政府管理方式，用改革创新的办法推动发展、化解矛盾、维护稳定。

切实加强廉政建设。坚持把纪律和规矩挺在前面，深入开展政府系统党风廉政建设和反腐败斗争，坚决惩治和查处损害群众利益的不正之风和腐败问题。严格落实中央八项规定精神、国务院“约法三章”和省市各项规定，自觉践行“三严三实”，坚持不懈狠刹“四风”。深入开展“两学一做”学习教育。牢固树立过紧日子思想，坚持开源节流、勤俭节约，严控“三公”经费。强化行政监察和审计监督，加强对重点领域、关键环节、重要岗位权力运行的监督制约。

不断提升服务水平。深化“简政放权、放管结合、优化服务”改革，进一步优化办事流程，提高办事效率。全面清理规范行政审批中介服务，降低行政成本，优化政务环境。推进政务公开，增强政府工作透明度。加强政府信息化建设，完善政府网上服务。强化为民意识和服务意识，创新公共服务方式，提升公共服务质量，让群众办事更方便、更快捷、更满意。

一分部署，九分落实。我们要始终牢记习近平总书记“有多大担当才能干多大事业，尽多大责任才能有多大成就”的教诲，大力弘扬“马上就办、真抓实干”的工作作风，大力倡导敢于负责、勇于创新的担当精神，永不懈怠、永不松劲，夙夜在公、勤勉工作，埋头苦干、精准发力，扎扎实实做好每一项工作，确保各项目标任务落到实处、收到实效。

各位代表，蓝图已经绘就，目标已经明确。让我们在省委、省政府和市委的坚强领导下，以更加饱满的热情、更加昂扬的斗志，凝神聚力，锐意进取，努力完成今年经济社会发展目标任务，确保实现“十三五”良好开局，为如期实现脱贫攻坚和全面建成小康社会目标而努力奋斗！
